# Modelling Training Adaptation in Swimming Using Artificial Neural Network Geometric Optimisation

**DOI:** 10.3390/sports8010008

**Published:** 2020-01-16

**Authors:** Justin Carrard, Petr Kloucek, Boris Gojanovic

**Affiliations:** 1 Doctoral School, Faculty of Biology and Medicine, University of Lausanne, 1015 Lausanne, Switzerland; 2 Division of Sports and Exercise Medicine, Department of Sport, Exercise and Health, University of Basel, 4052 Basel, Switzerland; 3 CAMPsyN, Hôpital de Cery, Lausanne University Hospital, 1008 Prilly, Switzerland; kloucek@me.com; 4 Sports Medicine, Swiss Olympic Medical Centre, Hôpital de La Tour, 1217 Meyrin, Switzerland; boris.gojanovic@latour.ch; 5 Sports Medicine, Swiss Olympic Medical Centre, Lausanne University Hospital, 1011 Lausanne, Switzerland

**Keywords:** training monitoring, online tool, machine learning

## Abstract

This study aims to model training adaptation using Artificial Neural Network (ANN) geometric optimisation. Over 26 weeks, 38 swimmers recorded their training and recovery data on a web platform. Based on these data, ANN geometric optimisation was used to model and graphically separate adaptation from maladaptation (to training). Geometric Activity Performance Index (GAPI), defined as the ratio of the adaptation to the maladaptation area, was introduced. The techniques of jittering and ensemble modelling were used to reduce overfitting of the model. Correlation (Spearman rank) and independence (Blomqvist β) tests were run between GAPI and performance measures to check the relevance of the collected parameters. Thirteen out of 38 swimmers met the prerequisites for the analysis and were included in the modelling. The GAPI based on external load (distance) and internal load (session-Rating of Perceived Exertion) showed the strongest correlation with performance measures. ANN geometric optimisation seems to be a promising technique to model training adaptation and GAPI could be an interesting numerical surrogate to track during a season.

## 1. Introduction

Training monitoring is widely understood to be a crucial part of modern athletes’ follow-up as it helps to assess individual response to training [1]. Consequently, training monitoring could also reduce the risk of overtraining syndrome, injury, illness, and eventually benefit athletic performance [1,2].

Several methods have been developed to quantify training load and assess fatigue and recovery [1,2,3]. The choice of adequate methods should depend on the sport-specific context, the goal of the monitoring program, as well as on available means and resources [1,4]. Once collected, data need to be analysed to provide coaches and athletes with actionable information [1,4,5]. To achieve this, several mathematical techniques to model training effects on performance have been proposed [6]. Traditional models, like impulse-response and multiple regression models, “are based on linear mathematical concepts such as regression analysis and linear differential equations” [6] (p. 839). However, because biological adaptations are complex non-linear processes, non-linear mathematical concepts like Artificial Neural Network (ANN) are believed to provide a more accurate description of performance responses to training [1,6,7,8,9,10]. Indeed, ANN models can capture dynamic changes in the training–performance relationship and adjust accordingly [11]. Furthermore, ANN is particularly appropriate to predict outcomes [12,13,14]. One study accurately predicted the performance of a single elite swimmer at the 2000 Olympic Games using ANN to model her training adaptation [8]. A second study demonstrated that using ANN to model training adaptation improved performance prediction in a triathlon, compared to a traditional linear model [15]. Finally, a third study reported improved performance prediction in cycling using a hybrid ANN ensemble model [11].

To date, however, to the best of the authors’ knowledge, no study has used ANN geometric optimisation to model training adaptation. Compared to ANN, ANN geometric optimisation can separate domains of influence of identified patterns in a Euclidean space [16,17]. In other words, it identifies patterns within a data set, determines the distribution of these patterns in a given space and finally detects the graphical boundaries of these patterns.

This study has two aims. The first is the monitoring of training, recovery and performance among competitive swimmers during 26 weeks. The second is the modelling of training adaptation using ANN geometric optimisation. It is hypothesised that ANN geometric optimisation could graphically separate adaptation from maladaptation (to training) and therefore facilitate outcome visualisation and comprehension for coaches and athletes.

Swimmers were selected as the target sample as they typically complete high training loads and are at higher risk of non-functional overreaching (NFOR) or overtraining (OT), compared to other athletes [18]. Studies report a NFOR/OT prevalence of about 10% in swimmers with career rates up to 30% [19,20]. NFOR/OT usually results from an imbalance between training load and everyday stressors on one hand, and adaptation and recovery capacity on the other hand [19]. Therefore, swimmers could benefit from an efficient training monitoring system [1]. 

## 2. Materials and Methods

### 2.1. Experimental Approach

The present explorative study used a prospective observational multicentre design to record training, recovery and performance parameters.

### 2.2. Recruitment

Coaches of all Swiss swim clubs recognised as “Talents Promotion Centres” were contacted by e-mail in the summer of 2013. Six of the 25 coaches were interested in participating. The study was presented to the swimmers of these six clubs, and they received an informative study protocol. A total of 39 athletes (20 males and 19 females, mean age 17.5 ± 2.8 years) agreed to participate and gave their informed written consent. Swimmers’ levels ranged from regional to international. The study was conducted in accordance with the Declaration of Helsinki and the protocol was approved on the 3rd of September 2014 by the Human Research Ethics Committee of the “Canton de Vaud” (Lausanne, Switzerland; study protocol no 321/13).

Since participants came from both the German- and the French-speaking parts of Switzerland, the study was conducted in both languages. The study observation period comprised 26 weeks during the first two macrocycles of the 2013–2014 season, from the 30th of September 2013 to the 30th of March 2014. When the study observation period started, swimming clubs had already been training for 4 weeks. 

### 2.3. Exclusion and Inclusion Criteria

All athletes wishing to participate in the study had to be a member of one of the participating swimming clubs. All clubs were recognised as “Talent Promotion Centres”, which certifies that all participants had at least a regional level, trained a minimum of 10 h per week and were at least 13 years old. Swimmers were excluded if an overtraining syndrome had been diagnosed during the previous season and/or they had an injury preventing participation in training at the start of the study. One swimmer had to be excluded for this reason. To avoid duplicates, the entire flow chart is illustrated in Figure 2 under Results.

### 2.4. Data Collection

Methods to quantify training load and assess fatigue and recovery were chosen according to recommendations made by Bourdon, et al. [1] and Soligard, et al. [2]. Recorded data and the pre-determined frequency at which they were recorded are summarised in Table 1 and described below. Throughout the study, swimmers entered data in a web platform (AthleteMonitoring, FITSTATS Technologies, Inc., Mancton, NB, Canada). Participants were instructed on how to use the platform during the study presentation. The platform automatically sent e-mail reminders if no data were entered on the previous day, as well as on days of questionnaire completion. The main investigator tracked all entered data daily and sent up to two additional reminders if necessary (on the day after, and again on the following day). Data were stored solely in anonymous form. Swimmers and coaches had neither feedback on, nor access to, the data entered during the study. 

#### 2.4.1. “Well-Being Questionnaire”

This questionnaire was developed by McLean et al. and slightly adapted to our needs [21]. Swimmers answered questions on five components of recovery: sleep quality and quantity, level of muscle soreness, training enjoyment and general stress (i.e. including stress outside training). Additionally, they assessed their global recovery level. A 7-point Likert scale was used, where one to three represent an insufficient, four an acceptable and five to seven a good level of recovery. All questions refer to the lapse of time since the last questionnaire was completed, except for sleep-related questions, which refer to the previous night.

#### 2.4.2. “Profile of Mood State—Adolescents (POMS-A)”

This POMS version consists of 24 items representing six mood dimensions: anger, confusion, depression, fatigue, tension and vigour. This version was chosen because most participants were teenagers [22,23]. Validated French and German translations were used for the adjectives, which are used in both the POMS-A and in the original POMS version [24,25]. The remaining adjectives were translated and examined by bilingual native speakers.

#### 2.4.3. Training Log

Coaches provided training attendance lists used to calculate the percentage of recorded training sessions. Based on the rating of perceived exertion (RPE), session-RPE, training monotony, training strain and Acute: Chronic Workload Ratio were calculated [26,27,28]. 

#### 2.4.4. Performance Outcome

Competition results were considered as a performance outcome and were collected from swimrankings (an online database for European swimmers). In order to judge whether a swimmer improves or not during the study, only her/his best discipline, defined as the one in which she/he has the most FINA points (in the year 2013) at the beginning of the study, was taken into account [29]. The achieved time was converted into “percentage of personal best time” (%PBT) as shown herein below (according to the formula used by swimrankings). Using the best time at the beginning of the study for the term “previous personal best time” allows for comparison of all competition results against the same performance index for each swimmer.

$$\begin{array}{c} Percentage\;of\;Personal\;Best\;Time\\ \;={\left(\frac{previous\;personal\;best\;time\left[seconds\right]}{current\;achieved\;time\left[seconds\right]}\right)}^{2}*100 \end{array}$$

### 2.5. Modelling Training Adaptation Using ANN Geometric Optimisation

#### 2.5.1. Concept

ANN geometric optimisation was used to model and graphically separate adaptation from maladaptation (to training). Weekly %PBT values were dichotomised by comparing each %PBT value against 100%. In this way, %PBT values exceeding 100% could stand out from %PBT values under 100% allowing easy separation of weeks with improvement (adaptation), vs. no improvement (maladaptation). 

#### 2.5.2. Mathematical Considerations Regarding the Development of the Model

Three different time series, X(t*_i_*), Y(t*_i_*), and Z(t*_i_*), *i* = 1,..., n, n ∈ Ν were projected on a three-dimensional Euclidean space using their Hausdorff-Besicovitch dimensions [30]. The time series Z(t*_i_*) was turned into a binary perceptron by comparing each value of the time series against 100%.

Based on the triplets (X(t*_i_*), Y(t*_i_*), sign(100 − Z(t*_i_*))), *i*=1,..., n, ANN was used to classify patterns that are characterised by respective perceptron’s values being either plus one or negative one. Mean Cross Entropy was used as a classifier [31]. The activation function is given by the perceptron. To locate the position and identify the domain of influence of these patterns, Calinski-Harabasz cluster criterion and three geometric techniques were used: Bray-Curtis dissimilarity, Chebyschev distance and normalized squared Euclidean distance [32,33,34,35,36]. The result with the least number of patterns was selected. Convexification of the respective patterns were computed as a coarse-grained partitioning of the domain of influence. These steps fundamentally simplify the subsequent construction of higher resolution Delaunay triangulations [37,38]. Lastly, the respective patterns’ domains of influence were disconnected by small layers to improve the integration error. 

**Figure 1:**
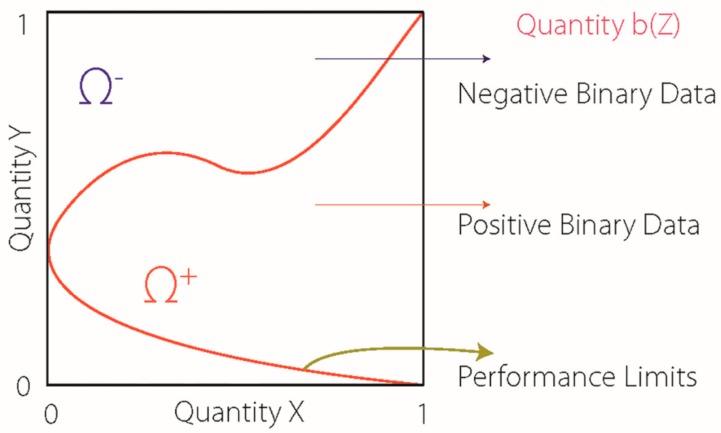
Artificial Neural Network geometric optimisation approach to monitoring. Ω^+^ = positive predictive performance area; Ω^−^ = negative predictive performance area; Quantity b(Z) = binary parameter.

The obtained output is a curve separating the domains of influence of identified patterns (Figure 1). In other words, constructed graphs display two areas: an adaptation and a maladaptation area. The software used to compute the model, “Cassiopee computational eco-system”, was developed by Kloucek [39]. 

#### 2.5.3. Inputs to the Model

Five different combinations of three time series were used to feed the model (Table 2). In each combination, %PBT was used as the time series Z(t*_i_*) and converted into the binary perceptron. This allows the contrast of the time series X(t*_i_*) and Y(t*_i_*), with the performance outcome. 

To choose the time series X(t*_i_*) and Y(t*_i_*), the authors classified collected data into coping and load, further classified as external and internal load (Table 3). In agreement with the recommendations of Bourdon, et al. [1], the combinations one and five include measures of external and internal load. Combinations two, three and four include measures of internal load and recovery as recommended by Kentta and Hassmen [40]. Due to financial constraints, this explorative research was limited to five out of the 44 possible combinations.

Weekly averages were used as time series X(t*_i_*) and Y(t*_i_*), while the best weekly %PBT was used as Z(t*_i_*). Data was normalised dividing each value of the time series by its maximal value. 

#### 2.5.4. Overfitting

Given its flexibility, ANN is prone to overfitting [11,15]. To reduce overfitting in models using small data sets, several approaches have been proposed [11]. Jittering “consists of adding artificial noise to data, thus supplementing the training sample with additional artificially created data which is similar to, but different from, the original data” [11] (p. 78). Ensemble modelling associates the output of several single models to improve the predictive performance of a single model and reduce overfitting [11,41]. There is no consensus for selecting the appropriate size of an ensemble, but an ensemble size of 5 to 10 is usually considered as sufficient [11,41,42,43]. In this study, 10% Gaussian white noise perturbation was superimposed to the original data sets creating new data sets [44]. For each swimmer and combination, 50 different data sets were created. Using these new data sets, the model was then run 50 times, for each swimmer and combination. The ensemble size of 50 was chosen arbitrarily. Simple averaging was then used to combine the obtained outputs [45].

#### 2.5.5. Goodness of Fit of the Model

Goodness of fit of the model was defined as the ratio of time-instances classified in the correct area regarding %PBT to time-instances classified in the wrong area according to %PBT.

#### 2.5.6. Geometric Activity Performance Index

The Geometric Activity Performance Index (GAPI) was introduced in an attempt to convert the geometrical output of the model into one single number and thus propose a potential numerical surrogate to track during a swimming season. GAPI is defined as the ratio of the adaptation to the maladaptation area. GAPI is directly proportional to the adaptation area (the larger this area, the higher the GAPI). To identify the GAPI which best correlates with the performance outcome, correlation (Spearman rank) and independence tests (Blomqvist β, also known as medial correlation coefficient) were run between GAPI of the five chosen combinations and both best %PBT and quartiles of best %PBT. Those tests were chosen because they are both non-parametric and appropriate for monotonic function. *p*-values ≤ 0.05 were considered statistically significant. Statistic- and *p*-values were rounded to two scientifically relevant decimals in accordance with the integration error. The Bonferroni-Holm method was used to control for the multiple comparisons problem [46].

#### 2.5.7. Prerequisites for the Modelling

Swimmers had to satisfy the following prerequisites in order to be included in the modelling: (1) >80% response rate in every questionnaire; (2) >75% of training sessions recorded; (3) at least one %PBT >100 % and one %PBT <100%; and (4) complete data set for all weeks having a %PBT value. The first two prerequisites ensure that swimmers entering into the analysis had a high level of compliance with the study protocol. The last prerequisite ensures that weeks without performance outcome were included in the analysis. Three coordinates (x; y; z) are required for each time-instance in order to compute such a graph. This reduced the number of time-instances to between four and eight per swimmer, which consequently also corresponds to the number of performances analysed per swimmer. 

## 3. Results

### 3.1. Swimmers’ Characteristics

**Figure 2:**
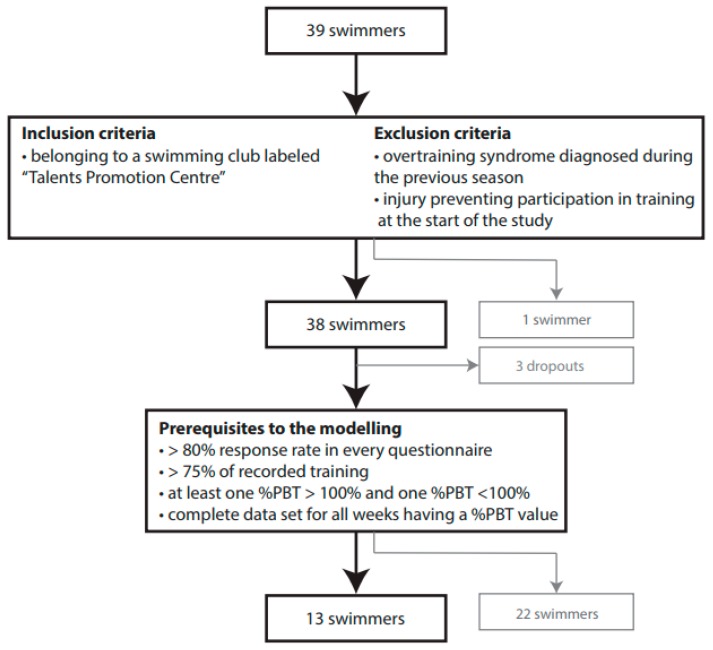
Participants flow. Abbreviations: %PBT = percentage of Personal Best Time.

From the initial cohort of 38 swimmers, three dropped out due to time restrictions or changing swimming clubs, and one retired from swimming as illustrated in Figure 2. Thirteen swimmers met the prerequisites to the modelling (Table 4). The seven swimmers whose %PBT values were exclusively over 100%, had an average age of 15.3 ± 2.1 years, and their mean best FINA points value at the beginning of the study was 462 ± 78. The six swimmers whose %PBT values were exclusively under 100%, had an average age of 20.2 ± 2.6 years, and their mean best FINA points value was 694 ± 88. A Welch’s t-Test was run to compare both groups and revealed a significant difference namely swimmers with PBT values >100% were younger (p-value = 0.005) and had a lower swimming level (p-value < 0.001). This may be due to the fact younger, less-experienced swimmers have more room for improvement compared to older swimmers.

### 3.2. Modelling Training Adaptation using ANN Geometric Optimisation

**Figure 3:**
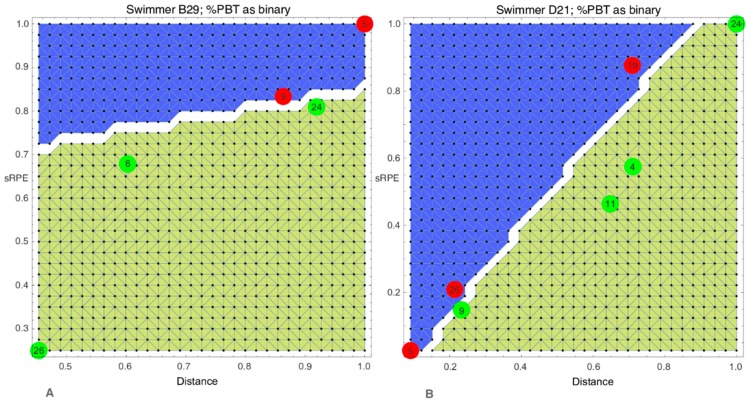
Modelling training adaptation using ANN geometric optimization. (**A**) Combination one “distance; sRPE; %PBT used as the binary separation parameter” based on ANN geometric optimisation for swimmer B29. (**B**) Combination one “distance; sRPE; %PBT used as the binary separation parameter” based on ANN geometric optimisation for swimmer D21. The yellow area represents the improvement area (i.e. %PBT > 100%) while the blue area represents the non-improvement area (i.e. %PBT < 100%). The white boundary represents the separation between them. Green dots represent weeks for which the %PBT is over 100%, while red dots represent weeks under 100%. Numbers in the dots represent week number. Misclassified dots are per definition red dots in the yellow area or green dots in the blue area (not illustrated here). They are due to the conditions we put on the optimisation to limit the number of separated regions. Abbreviations: %PBT = percentage of Personal Best Time, sRPE = session-RPE. As data was normalised dividing each value of the time series by its maximal value, there is no unit for x- and y-axis.

Figure 3 presents two graphs, obtained using ANN geometric optimisation. 

#### 3.2.1. Goodness of Fit of the Model

As indicated in Table 5, goodness of fit of the model was on average 95%.

#### 3.2.2. Geometric Activity Performance Index

After correcting the *p*-values with the Bonferroni-Holm method, GAPI of the first (distance and session-RPE), and to a lesser extent, fourth (training monotony and recovery) combinations were positively and significantly correlated with quartiles of best %PBT and best %PBT for both Spearman rank and Blomqvist β as shown in Table 6. GAPI of the combinations two (session-RPE and recovery), three (training strain and recovery) and five (distance and training stress-balance) did not show significant correlation with performance measures. 

## 4. Discussion

To the best of the authors’ knowledge, this is the first time a study has sought to model training adaptation using ANN geometric optimisation. 

The suggested model permitted the systematic tracking of athletes during the course of the season and allowed for a determination of whether a swimmer is located in the adaptation or maladaptation area, based on the learning process of ANN geometric optimisation. Moreover, it could provide clues to athletes and coaches regarding which variables need to be modified to reach the adaptation area. For example, in Figure 3A, week nine is situated in the maladaptation area; in order to move to the adaptation area, it can be concluded, based on this graphic representation, that training load should be reduced. Furthermore, for both swimmers in Figure 3A,B, the yellow and blue areas correspond to the areas for which session-RPE is less than distance, and session-RPE is greater than distance, respectively. This corresponds to hypotheses previously described in the literature, namely that internal training load < external training load indicates adaptation while internal training load > external training load indicates maladaptation (to training) [1]. 

GAPI of the first (distance and session-RPE), and to a lesser extent, fourth (training monotony and recovery) combinations were positively correlated with performance measures. Since GAPI is a single number, it could be an interesting surrogate to track during a swimming season. As long as swimmers have %PBT values both above and under 100%, this model could be used. If this is not the case, each %PBT value could be compared to the mean of %PBT values rather than to 100%. A model using 100% highlights absolute improvement, while a model using the mean of %PBT values highlights relative improvement. This model makes intra-individual and not inter-individual comparisons. Lastly, the principle of this model should be transferable to other sports but the collected data might need to be different according to the sport-specific context.

### 4.1. Strengths and Limitations

Several challenges were encountered in initiating this study. First, it was difficult to find coaches and swimmers interested in taking part in the study. Secondly, even when the web platform facilitated data collection, the main investigator had to track data entered on a daily basis and remind swimmers to enter data to increase compliance. Thirdly, the collaboration between the mathematician and sports and exercise physician provided further challenges due to the lack of overlay in their specialities.

In the field of machine learning, a model is considered as valid if it can generalise from training data set to unseen data [47]. Due to the small data set used in the present study, it was not possible to split it into separate training and validation data sets. A second reason for the inability of the authors to validate the model resides in the lack of funding for the study. It must be acknowledged that ANN is an expensive and time-consuming process [48]. Therefore, it is currently unclear whether the present model is valid or not. Consequently, validation of the present model is required before practical application.

If ANN is believed to model training adaptation more accurately than traditional linear models, and to be particularly appropriated for prediction, this technique has some limitations [8,14]. First, ANN is considered as a “black box” due to its inability to identify causal relationships between input and output [11,49]. Thus, the interpretation of the obtained results might be more complex. Secondly, the more data ANN receives, the more it learns [50]. However, small data sets are often inevitable in training monitoring [11]. Indeed, “training data accumulates with one data point per training, per athlete”, while competition does not occur every week in sports like swimming [11] (p. 66). Therefore, techniques such as jittering and ensemble modelling are needed to overcome issues related to small data sets [11]. With a number of competition results ranging from four to eight, the precision with which the adaptation and maladaptation areas were constructed might be improved with more data. Retrospectively, having chosen ANN to model training adaptation in the present study might not have been the best choice in the light of the small data set, absence of funding and subsequent inability to validate the model.

The next limitation is that this model is currently based on only two variables at a time. Whilst it is clear that more than two variables contribute to training adaptation, this work attempts to develop an easy-to-use tool able to estimate training adaptation rather than to predict performance in the most accurate way. In theory, it would be possible to include more variables by computing n-dimensional graphs. However, such graphs will be far less readable. Another way to include more variables could be to use a score of different data rather than a single datum as coordinates.

Interestingly, another limitation of ANN turned into a strength in this case, namely that for ANN there is no one-size-fits-all solution [51]. This nicely fits the need for individualisation of training and recovery monitoring [1,3]. Another strength of using ANN relies on its ability to learn from existing data to create future datasets. This is highly interesting in training science to inform coaches and athletes early about training efficiency.

### 4.2. Future Studies

A validation attempt using unseen data is required before any practical application of the present model. It would then be very interesting to know how long and how far athletes could go into the maladaptation area without suppressing the super compensation mechanism or suffering from NFOR/OT. It is an accepted principle of training that athletes temporarily leave the adaptation for the maladaptation area; therefore, adaptation modelling could provide important input into the balance between time spent in both areas [52].

## 5. Conclusions

Due to the fact that the present model has not been validated, results should be considered as preliminary and interpreted with caution. Artificial Neural Networks geometric optimisation seems to be a promising technique to model individual training adaptation during a season, separating adaptation to maladaptation (to training), at any given week. Additionally, this model might provide coaches and athletes with graphical clues on how to reach the adaptation area. The Geometrical Activity Performance Index (GAPI) could be an interesting numerical surrogate to track during a season. The GAPI based on external load (distance) and internal load (session-RPE) showed the strongest correlation with performance measures. 

## Figures and Tables

**Table 1:** Recorded data. Abbreviation: The POMS-A = The Profile of Mood State—Adolescents.

Frequency	Data Type	Reminders
**Daily** After every training session	Training log: Rating of perceived exertion (RPE) using the modified Borg CR-10 RPE scale Sport type Distance (meters, if swimming) Duration (minutes)	If no training was entered on the previous day, the web platform automatically sent a reminder email on the following day.
**Twice a week** Every Tuesday and Friday	The Well-being questionnaire	An email was sent on the day of completion and if needed up to two additional reminders were sent (on the day after and on the day after next).
**Fortnightly** Every second Sunday	The POMS-A	An email was sent on the day of completion and if needed up to two additional reminders were sent (on the day after and on the day after next).

**Table 2:** The five combinations analysed. Abbreviations: %PBT = percentage of Personal Best Time.

Time Series ►	x	y	z
Combinations ▼			
**1**	Distance	Session-RPE	%PBT (binary)
**2**	Session-RPE	Recovery	%PBT (binary)
**3**	Training strain	Recovery	%PBT (binary)
**4**	Training monotony	Recovery	%PBT (binary)
**5**	Distance	Acute: Chronic Workload Ratio	%PBT (binary)

► refers to this first row, while ▼refers to the first column.

**Table 3:** Classification of the recorded parameters.

Load Parameters		Coping Parameters
External Load	Internal Load	
Distance	Session-RPE Training strain Training monotony Acute:Chronic Workload Ratio	Recovery Sleep quality Sleep quantity Soreness Pleasure Stress Total Mood Disturbance

Abbreviation: RPE = Rating of Perceived Exertion.

**Table 4:** Individual swimmer’s characteristics.

Swimmer	Sex	Age (Year)	Quartile	Best Discipline (meter)	FINA Points 2013	Quartile	Best %PBT	Quartile	Weekly Mean Internal Training Load (AU)	Quartile	Weekly Mean Distance (meter)	Quartile
A2	♂	18	3	400 freestyle ld	765	4	100.1	1	4258.46	4	30,100	4
B5	♀	14	1	200 breaststroke ld	633	3	110	4	3144.23	3	18,826.92	2
B6	♀	15	1	100 freestyle sd	459	1	100.7	1	2775	2	17,148	2
B29	♀	15	1	50 breaststroke ld	504	1	105.9	3	2377.31	1	15,426.92	1
C10	♂	19	4	100 medley sd	582	2	103.1	2	2504.81	1	13,905.77	1
C13	♂	16	2	100 freestyle ld	471	1	103.7	3	3353.08	3	23,386.54	3
C14	♂	16	2	400 medley ld	445	1	109.2	4	2365.38	1	16,350	1
D21	♀	15	1	400 freestyle ld	640	3	106.1	4	4417.71	4	27,253.33	4
D22	♀	15	1	200 breaststroke ld	617	2	102.4	1	2946.4	2	32,212.8	4
D35	♀	18	3	100 freestyle ld	631	3	100.7	1	2850.38	2	25,732.69	3
E24	♂	19	4	100 freestyle ld	646	4	104.9	3	2112.5	1	15,411.46	1
E27	♀	18	3	100 backstroke ld	619	2	102.7	2	4703.27	4	23,744.23	3
E28	♂	20	4	50 butterfly ld	673	4	103.0	2	3128.46	3	22,900	2

Abbreviations: ♂= male, ♀= female, ld = long distance, sd = short distance, AU = Arbitrary Unit, Best %PBT = best performance during the study expressed in percentage of personal best time; quartiles always refer to the parameter of the previous column and serve to compare a given swimmer with the other swimmers.

**Table 5:** Goodness of fit of the model (in %).

Combinations ►	1	2	3	4	5
Swimmers▼					
**A2**	75	100	100	100	100
**B5**	88	75	88	88	88
**B6**	100	100	83	100	100
**B29**	100	80	80	100	100
**C10**	100	100	100	100	100
**C13**	100	100	100	100	100
**C14**	100	100	100	100	75
**D21**	100	100	100	100	86
**D22**	100	100	100	86	100
**D35**	100	100	100	100	100
**E24**	100	100	100	100	100
**E27**	100	100	100	100	100
**E28**	75	63	88	88	75
**Average**	95	94	95	97	94
**Global average**	95				

► refers to this first row, while ▼refers to the first column.

**Table 6:** Correlation tests between GAPI and the improvement quartile/best %PBT.

	Correlation Tests	Quartile			Best %PBT		
		Statistic	Original *p*-Value	Corrected *p*-Value	Statistic	Original *p*-Value	Corrected *p*-Value
**GAPI_1**	Spearman rank	0.85	<0.01	<0.01	0.85	<0.01	<0.01
	Blomqvist β	0.93	<0.01	<0.01	0.92	<0.01	<0.01
**GAPI_2**	Spearman rank	0.35	0.25	0.51	0.33	0.28	0.28
	Blomqvist β	0.46	0.25	0.75	0.42	0.08	0.32
**GAPI_3**	Spearman rank	0.56	0.05	0.13	0.59	0.03	0.13
	Blomqvist β	0.46	0.25	0.25	0.42	0.08	0.16
**GAPI_4**	Spearman rank	0.62	0.02	0.02	0.65	0.01	0.04
	Blomqvist β	0.74	0.02	0.03	0.67	<0.01	<0.01
**GAPI_5**	Spearman rank	0.39	0.20	0.40	0.47	0.10	0.31
	Blomqvist β	0.46	0.25	0.25	0.42	0.08	0.32

Abbreviations: GAPI_n = GAPI of the nth correlation. %PBT = percentage of Personal Best Time.

## Data Sharing Statement

Raw data are available on request to the corresponding author.

## References

[1] Bourdon P.C., Cardinale M., Murray A., Gastin P., Kellmann M., Varley M.C., Gabbett T.J., Coutts A.J., Burgess D.J., Gregson W. (2017). Monitoring athlete training loads: Consensus statement. Int. J. Sports Physiol. Perform..

[2] Soligard T., Schwellnus M., Alonso J.M., Bahr R., Clarsen B., Dijkstra H.P., Gabbett T., Gleeson M., Hagglund M., Hutchinson M.R. (2016). How much is too much? (part 1) international olympic committee consensus statement on load in sport and risk of injury. Br. J. Sports Med..

[3] Halson S.L. (2014). Monitoring training load to understand fatigue in athletes. Sports Med..

[4] Gabbett T.J., Nassis G.P., Oetter E., Pretorius J., Johnston N., Medina D., Rodas G., Myslinski T., Howells D., Beard A. (2017). The athlete monitoring cycle: A practical guide to interpreting and applying training monitoring data. Br. J. Sports Med..

[5] Foster C. (2019). Sport science: Progress, hubris, and humility. Int. J. Sports Physiol. Perform..

[6] Jobson S.A., Passfield L., Atkinson G., Barton G., Scarf P. (2009). The analysis and utilization of cycling training data. Sports Med..

[7] Pfeiffer M., Hohmann A. (2012). Applications of neural networks in training science. Hum. Mov. Sci..

[8] Edelmann-Nusser J., Hohmann A., Henneberg B. (2002). Modeling and prediction of competitive performance in swimming upon neural networks. Eur. J. Sport Sci..

[9] Fister I., Ljubič K., Suganthan P.N., Perc M., Fister I. (2015). Computational intelligence in sports: Challenges and opportunities within a new research domain. Appl. Math. Comput..

[10] Balagué N., Torrents C. (2005). Thinking before computing: Changing approaches in sports performance. Int. J. Comput. Sci. Sport.

[11] Churchill T. (2014). Modelling Athletic Training and Performance: A Hybrid Artificial Neural Network Ensemble Approach. PhD in Information Sciences and Engineering. Ph.D. Thesis.

[12] Hellard P., Avalos M., Lacoste L., Barale F., Chatard J.-C., Millet G.P. (2006). Assessing the limitations of the banister model in monitoring training. J. Sports Sci..

[13] Bunker R.P., Thabtah F. (2019). A machine learning framework for sport result prediction. Appl. Comput. Inf..

[14] Samarasinghe S. (2016). Neural Networks for Applied Sciences and Engineering: From Fundamentals to Complex Pattern Recognition.

[15] Haar B. (2011). Analyse und Prognose von Trainingswirkungen: Multivariate Zeitreihenanalyse Mit Künstlichen Neuronalen Netzen. Analysis and Prediction of Training Effects: Multivariate Time Series Analysis with Artificial Neural Networks. Ph.D. Thesis.

[16] Bishop C.M., Tipping M.E. (1998). A hierarchical latent variable model for data visualization. IEEE Trans. Pattern Anal. Mach. Intell..

[17] Tipping M.E. (2001). Sparse bayesian learning and the relevance vector machine. J. Mach. Learn. Res..

[18] Khodaee M., Edelman G.T., Spittler J., Wilber R., Krabak B.J., Solomon D., Riewald S., Kendig A., Borgelt L.M., Riederer M. (2016). Medical care for swimmers. Sports Med. Open.

[19] Meeusen R., Duclos M., Foster C., Fry A., Gleeson M., Nieman D., Raglin J., Rietjens G., Steinacker J., Urhausen A. (2013). Prevention, diagnosis, and treatment of the overtraining syndrome: Joint consensus statement of the european college of sport science and the american college of sports medicine. Med. Sci. Sports Exerc..

[20] Matos N.F., Winsley R.J., Williams C.A. (2011). Prevalence of nonfunctional overreaching/overtraining in young english athletes. Med. Sci. Sports Exerc..

[21] McLean B.D., Coutts A.J., Kelly V., McGuigan M.R., Cormack S.J. (2010). Neuromuscular, endocrine, and perceptual fatigue responses during different length between-match microcycles in professional rugby league players. Int. J. Sports Physiol. Perform..

[22] Terry P.C., Lane A.M., Lane H.J., Keohane L. (1999). Development and validation of a mood measure for adolescents. J. Sports Sci..

[23] Terry P.C., Lane A.M., Fogarty G.J. (2003). Construct validity of the profile of mood states—adolescents for use with adults. Psychol. Sport Exerc..

[24] Cayrou S., Dickès P., Dolbeault S. (2003). Version française du profile of mood states (poms-f). The french version of the profile of mood states (poms-f). J. Thér. Comport. Cogn..

[25] Albani C., Blaser G., Geyer M., Schmutzer G., Brahler E., Bailer H., Grulke N. (2005). Überprüfung der gütekriterien der deutschen kurzform des fragebogens profile of mood states (poms) in einer repräsentativen bevölkerungsstichprobe. The german short version of profile of mood states (poms): Psychometric evaluation in a representative sample. Psychother. Psychosom. Med. Psychol..

[26] Foster C. (1998). Monitoring training in athletes with reference to overtraining syndrome. Med. Sci. Sports Exerc..

[27] Wallace L.K., Slattery K.M., Coutts A.J. (2009). The ecological validity and application of the session-rpe method for quantifying training loads in swimming. J. Strength Cond. Res..

[28] Gabbett T.J. (2016). The training-injury prevention paradox: Should athletes be training smarter and harder?. Br. J. Sports Med..

[29] Fédération Internationale de Natation (FINA), Fina Points. http://www.fina.org/content/fina-points.

[30] Badii R., Politi A. (1984). Hausdorff dimension and uniformity factor of strange attractors. Phys. Rev. Lett..

[31] De Boer P.-T., Kroese D.P., Mannor S., Rubinstein R.Y. (2005). A tutorial on the cross-entropy method. Ann. Oper. Res..

[32] Caliński T., Harabasz J. (1974). A dendrite method for cluster analysis. Commun. Stat. Theory Methods.

[33] Greenacre M. (2017). Ordination with any dissimilarity measure: A weighted euclidean solution. Ecology.

[34] Van Cutsem B. (2012). Classification and Dissimilarity Analysis.

[35] Cantrell C.D. (2000). Modern Mathematical Methods for Physicists and Engineers.

[36] Krebs C.J. (1989). Ecological Methodology.

[37] Lee D.-T., Schachter B.J. (1980). Two algorithms for constructing a delaunay triangulation. Int. J. Comput. Inf. Sci..

[38] Field D.A. (1988). Laplacian smoothing and delaunay triangulations. Commun. Appl. Numer. Methods.

[39] Kloucek P. Cassiopée Applied Analytical Systems. http://www.cassiopee.org/index.html.

[40] Kentta G., Hassmen P. (1998). Overtraining and recovery. A conceptual model. Sports Med..

[41] Perrone M., Cooper L. (1993). When networks disagree: Ensemble methods for hybrid neural networks. Neural Netw. Speech Image Process..

[42] Tufféry S. (2011). Data Mining and Statistics for Decision Making.

[43] Zhang G.P. (2007). A neural network ensemble method with jittered training data for time series forecasting. J. Inf. Sci..

[44] Kuo H.-H. (2018). White Noise Distribution Theory.

[45] Sharkey A.J.C. (1996). On combining artificial neural nets. Connect. Sci..

[46] Aickin M., Gensler H. (1996). Adjusting for multiple testing when reporting research results: The bonferroni vs holm methods. Am. J. Public Health.

[47] Poggio T., Rifkin R., Mukherjee S., Niyogi P. (2004). General conditions for predictivity in learning theory. Nature.

[48] Expensive, Labour-Intensive, Time-Consuming: How Researchers Overcome Barriers in Machine Learning. https://medium.com/@1nst1tute/expensive-labour-intensive-time-consuming-how-researchers-overcome-barriers-in-machine-learning-4f686b2a1979.

[49] Hellard P., Avalos M., Guimaraes F., Toussaint J.F., Pyne D.B. (2015). Training-related risk of common illnesses in elite swimmers over a 4-yr period. Med. Sci. Sports Exerc..

[50] Cain G. (2017). Artificial Neural Networks: New Research.

[51] Olawoyin A., Chen Y. (2018). Predicting the future with artificial neural network. Procedia Comput. Sci..

[52] Issurin V.B. (2010). New horizons for the methodology and physiology of training periodization. Sports Med..

